# Machine learning-based improvement of an online rheumatology referral and triage system

**DOI:** 10.3389/fmed.2022.954056

**Published:** 2022-07-22

**Authors:** Johannes Knitza, Lena Janousek, Felix Kluge, Cay Benedikt von der Decken, Stefan Kleinert, Wolfgang Vorbrüggen, Arnd Kleyer, David Simon, Axel J. Hueber, Felix Muehlensiepen, Nicolas Vuillerme, Georg Schett, Bjoern M. Eskofier, Martin Welcker, Peter Bartz-Bazzanella

**Affiliations:** ^1^Department of Internal Medicine 3, Friedrich-Alexander-University Erlangen-Nürnberg and Universitätsklinikum Erlangen, Erlangen, Germany; ^2^Deutsches Zentrum für Immuntherapie (DZI), Friedrich-Alexander-University Erlangen-Nürnberg and Universitätsklinikum Erlangen, Erlangen, Germany; ^3^Université Grenoble Alpes, AGEIS, Grenoble, France; ^4^Machine Learning and Data Analytics Lab, Department of Artificial Intelligence in Biomedical Engineering (AIBE), Friedrich-Alexander-Universität Erlangen-Nürnberg (FAU), Erlangen, Germany; ^5^Medizinisches Versorgungszentrum Stolberg, Stolberg, Germany; ^6^Klinik für Internistische Rheumatologie, Rhein-Maas-Klinikum, Würselen, Germany; ^7^RheumaDatenRhePort (rhadar), Planegg, Germany; ^8^Praxisgemeinschaft Rheumatologie-Nephrologie, Erlangen, Germany; ^9^Medizinische Klinik 3, Rheumatology/Immunology, Universitätsklinikum Würzburg, Würzburg, Germany; ^10^Verein zur Förderung der Rheumatologie e.V., Würselen, Germany; ^11^Division of Rheumatology, Klinikum Nürnberg, Paracelsus Medical University, Nürnberg, Germany; ^12^Faculty of Health Sciences, Center for Health Services Research, Brandenburg Medical School Theodor Fontane, Rüdersdorf, Germany; ^13^Institut Universitaire de France, Paris, France; ^14^LabCom Telecom4Health, Orange Labs and Univ. Grenoble Alpes, CNRS, Inria, Grenoble INP-UGA, Grenoble, France; ^15^MVZ für Rheumatologie Dr. Martin Welcker GmbH, Planegg, Germany

**Keywords:** artificial intelligence, machine learning, rheumatology, triage, symptom checker, digital health, decision support system (DSS)

## Abstract

**Introduction:**

Rheport is an online rheumatology referral system allowing automatic appointment triaging of new rheumatology patient referrals according to the respective probability of an inflammatory rheumatic disease (IRD). Previous research reported that Rheport was well accepted among IRD patients. Its accuracy was, however, limited, currently being based on an expert-based weighted sum score. This study aimed to evaluate whether machine learning (ML) models could improve this limited accuracy.

**Materials and methods:**

Data from a national rheumatology registry (RHADAR) was used to train and test nine different ML models to correctly classify IRD patients. Diagnostic performance was compared of ML models and the current algorithm was compared using the area under the receiver operating curve (AUROC). Feature importance was investigated using shapley additive explanation (SHAP).

**Results:**

A complete data set of 2265 patients was used to train and test ML models. 30.5% of patients were diagnosed with an IRD, 69.3% were female. The diagnostic accuracy of the current Rheport algorithm (AUROC of 0.534) could be improved with all ML models, (AUROC ranging between 0.630 and 0.737). Targeting a sensitivity of 90%, the logistic regression model could double current specificity (17% vs. 33%). Finger joint pain, inflammatory marker levels, psoriasis, symptom duration and female sex were the five most important features of the best performing logistic regression model for IRD classification.

**Conclusion:**

In summary, ML could improve the accuracy of a currently used rheumatology online referral system. Including further laboratory parameters and enabling individual feature importance adaption could increase accuracy and lead to broader usage.

## Introduction

Rheumatology services are facing an increasing demand of referrals while the number of rheumatologists is steadily declining (1). This shortage of rheumatologists results in a long delay for patients from symptom onset to rheumatology appointment, diagnosis and start of therapy. To reduce irreversible damage caused by uncontrolled inflammatory disease, rheumatologists have to triage patients. Despite various triage and screening strategies (2), the large majority of patients referred to rheumatologists end up not having an inflammatory rheumatic disease (IRD) (3, 4). In contrast to emergency medicine (5), no objective, rheumatology triage criteria exists and this lack of standardized triage decisions hampers quality of care. The European Alliance of Associations for Rheumatology (EULAR) recently emphasized the added value of patient preassessment by telehealth to improve the referral process to rheumatology and help prioritization of people with suspected IRD (6).

Rheport is an online rheumatology referral system, currently used in Germany to automatically triage appointments of new rheumatology patient referrals according to the respective probability of an IRD (7). An objective, weighted sum score is used to calculate the individual IRD probability. The tool can be used by patients themselves or referring physicians. Recent studies showed that Rheport was well accepted and perceived as easy to use by patients (8), however, the diagnostic accuracy was limited (4). Machine learning has been successfully used in various disciplines to increase diagnostic accuracy (9–11). In rheumatology, expert-level performance has recently been achieved using deep learning for detection of radiographic sacroiliitis (12) and individual risk of disease flares could be predicted in patients with rheumatoid arthritis using advanced machine learning (10). To our knowledge, no study has investigated the potential of machine learning to improve the triage of patients referred to rheumatology centers.

The aim of this study therefore was to evaluate whether machine learning could improve the triage accuracy (detection of inflammatory rheumatic diseases) of the online rheumatology self-referral system Rheport.

## Materials and methods

### Rheport questionnaire

Rheport^1^ was implemented at seven German rheumatology centers to automatically triage appointments of new rheumatology patient referrals according to the respective probability of an inflammatory rheumatic disease (IRD). The fixed Rheport patient questionnaire used in this study (version 1.0), consists of 23 questions, including basic health information, typical rheumatic symptoms, and laboratory parameters [C-reactive protein (CRP), erythrocyte sedimentation rate (ESR)]. Every question is assigned a weight in percentage and every possible answer is assigned a separate factor. All weights and factors are based on expert knowledge from rheumatologists. The sub-score of a question is calculated by multiplying the given answers factor by the weight of the question. Adding up all sub-scores of the 23 questions results in the total score of the patient. An example of a completed questionnaire is presented in Table 1.

**TABLE 1:** Example of a questionnaire summary report including the respective score calculation.

Question	Answers	Factor	Weight	Sub-score
Gender	Female	0	0%	0.00
Age	<60 years	0	2%	0.00
Weight loss	No	0	3%	0.00
Duration of complaints	6 months, <12 months	3	5%	0.15
Preceding injury	No	5	1%	0.05
Preceding infection	No	0	1%	0.00
Preceding tick sting	No	0	0%	0.00
Referring physician	General practitioner	0	1%	0.00
Lab results	No lab results available	0	10%	0.00
Family history	No	0	0%	0.00
Joint pain	With movement	1	10%	0.10
Joint swelling	Big toe	10	10%	1.00
Finger swelling	Whole finger	10	4.5%	0.45
Duration of joint swelling	>6 weeks, <half a year	10	2.5%	0.25
Joint stiffness	All day long	1	7.5%	0.075
Headache	Neck and back of head	1	7.5%	0.075
Lower back pain	No	0	7.5%	0.00
Other pain	No	0	7.5%	0.00
Pain related limitation of movement	No	0	2.5%	0.00
Muscle weakness	No	0	10%	0.00
General symptoms	Disrupted sleep, often tired	0	2.5%	0.00
Other symptoms	Fever >38°C	4	2.5%	0.10
Comorbidities	Psoriasis	4	2.5%	0.10
	Total score			2.35

Rheport consists of 4 different triage levels, based on total score thresholds (Table 2). Total scores lower 1 are classified as unlikely to have an IRD (non-IRD) and these patients are transferred (back) to their treating general physician. Patients with a minimum total score of 1 may book an appointment, being classified as IRD, at a participating rheumatology center. With increasing total scores patients have access to earlier appointments. The cut-offs were established after reviewing scores of the first 255 consecutive patients. IRD patients with scores < 1 (8/255) presented with very mild symptoms and later presentation would not have led to a decisive deterioration in prognosis in these case. Based on this cut-off, Rheport had a negative predictive value (NPV) of 86.4% and a positive predictive value (PPV) of 34.2%.

**TABLE 2:** Rheport’s triage levels, respective total score thresholds and appointment time frame.

Triage levels		Total score thresholds	Appointment time frame
1	Very urgent	>4.0	Within 1 week
2	Urgent	2.4–4.0	Within 2 weeks
3	Intermediate	1.0–2.4	Within 1 month
4	IRD unlikely	<1	Transfer (back) to GP

### Rheport dataset and data pre-processing

Prior to completing the questionnaire, users need to register and actively consent that their data is uploaded pseudonymized to the RheumaDatenRhePort (RHADAR) registry (7). RHADAR is a German real-world rheumatology registry including adult patients. Participating rheumatologists are encouraged to add their final diagnosis to the RHADAR registry. We only included fully completed questionnaires with matched added final diagnosis by the treating rheumatologist. Patients having consented until August 17th, 2020, were included in this analysis. Descriptive statistical analyses were carried out using to describe gender, age, inflammatory marker-, and family history-status, based on patient questionnaires and final diagnosis according to treating rheumatologist (Table 3).

**TABLE 3:** Diagnostic categories.

Diagnostic categories *n*, (%)	
Inflammatory rheumatic disease	690 (30.5)
Rheumatoid arthritis	339 (15.0)
Psoriatic arthritis	103 (4.5)
Polymyalgia rheumatica	84 (3.7)
Axial spondyloarthritis	56 (2.5)
Undifferentiated arthritis	44 (1.9)
Reactive arthritis	21 (0.9)
Systemic lupus erythematosus	18 (0.8)
Crystal arthropathies	12 (0.5)
Systemic sclerosis	5 (0.2)
Inflammatory idiopathic myositis	3 (0.1)
Behcet’s disease	2 (0.1)
ANCA-associated vasculitis	2 (0.1)
Giant cell arteritis	1 (0.04)
Non-inflammatory rheumatic disease	1,575 (69.5)

Most ML-models work with numerical data only (13). All the features in the dataset were non-numerical and had to be transcribed. For ordinal features (CRP, ESR, duration of joint swelling and joint complaints) a value was assigned for every category. For the remaining nominal features one-hot-encoding was used. For every category of the feature, a new dummy column was created. The entries in these columns were either 0 or 1.

To address the unequal IRD distribution in the dataset [and general population referred to rheumatologists (3, 4)], we used the Python library imbalanced-learn (14). This library provides different methods for over- and undersampling during training and testing of the ML models, such as SMOTE (15).

The dataset was used for training nine different ML-models, applying the Python library scikit-learn (16) including: K-nearest neighbor, decision tree, support vector machine, neural network, logistic regression, random forest, bagging classifier, gradient boosting, and AdaBoost.

### Model training, testing, and feature importance analysis

Using a validation dataset and setting it aside to test the model after training, results in less training data. Cross-validation avoids this problem and we therefore used a threefold cross-validation approach, where 2 parts acted as the training data while 1 part was used for testing. This was done 3 times, so that every part of the data once acted as the validation data. The prediction error is the average of the 3 computed errors. Cross-validation is also used for hyper-parameter tuning. A combination of both is called nested cross-validation. Without nested cross-validation, the same data that would be used for tuning the hyper-parameters and to estimate the model performance, which might result in overfitting. To avoid this, nested cross-validation uses an inner loop for tuning the hyper-parameters and an outer loop for generating the prediction error. The best performing model was selected based on the mean area under the receiver operating characteristics curve (AUC).

Diagnostic accuracy for the current algorithm and best performing model was further evaluated referring to sensitivity, specificity, NPV. Different clinical target sensitivities were explored (90 and 95%). For the best performing ML model, feature importance was investigated using shapley additive explanation (SHAP) (17, 18). This analysis indicates to which extent and in which direction (pro IRD vs. against IRD) a certain feature influences the ML model.

We used the TRIPOD checklist (19) (Supplementary Material 1) to enable transparent reporting of our study results.

## Results

### Patient dataset

**FIGURE 1:**
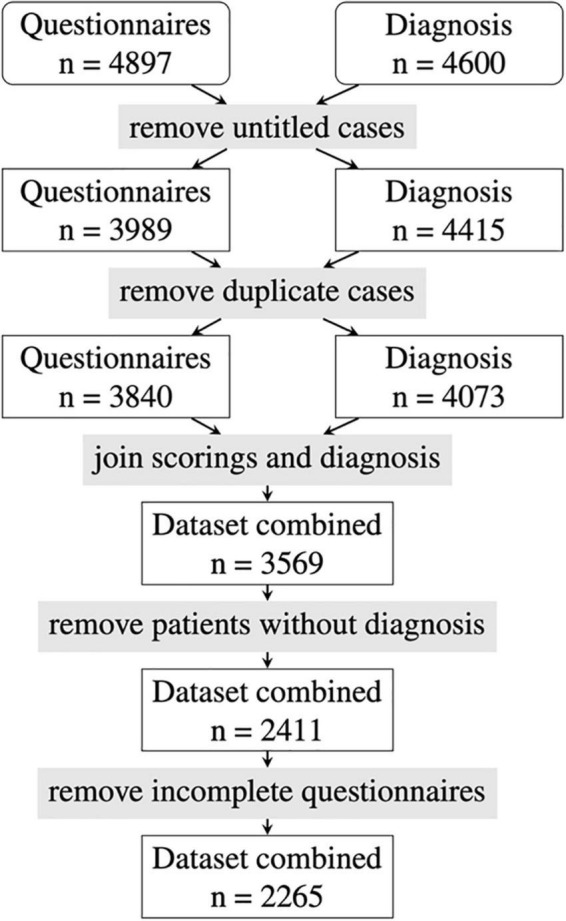
Flowchart of patient case selection.

A dataset of 4,897 Rheport questionnaires from seven German rheumatology centers with verified diagnosis was available. After removing invalid data (test vignettes, untitled cases) and duplicate data, another 1,158 patients with missing final diagnosis and 146 incomplete questionnaires had to be removed. The final dataset consisted of 2,265 patients (see Figure 1).

Table 3 displays the final diagnostic categories according to the treating rheumatologists. 690/2,265 (30.5%) of the patients were diagnosed with an IRD, the most common IRD being rheumatoid arthritis 339/690 (49.1%). Self-reported patient characteristics according to the Rheport questionnaire are listed in Supplementary Material 2. The majority of patients were female (69.3%) and younger than 60 years of age (76.9%). Only a minority reported that symptoms started less than 6 weeks ago (9.0%). The majority of patients had previously seen a general physician (85.1%). Only a minority of patients 839/2,265 (37.0%) entered lab results (CRP, ESG) completing the Rheport questionnaire. The large majority of patients complained of being tired (80.0%). The most commonly cited comorbidities were osteoarthritis (37.2%) and obesity (31.5%).

### Performance of machine learning models

**FIGURE 2:**
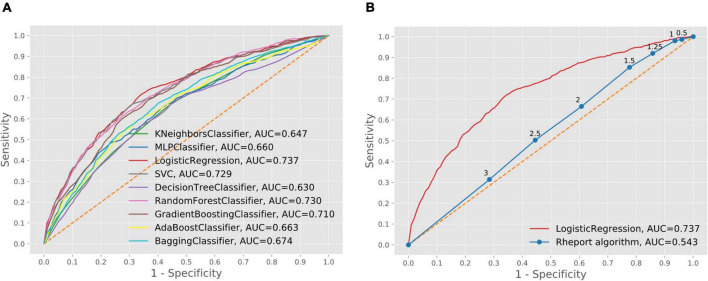
Comparison of machine learning model performance **(A)** and comparison of best performing machine learning model and current Rheport algorithm **(B)**.

The current Rheport algorithm (AUROC of 0.534) could be improved with all ML models, starting with decision tree (AUROC of 0.630) to the three best performing models, including support vector machine (AUROC of 0.729), random forest (AUROC of 0.730) and logistic regression (AUC of 0.737) (Figure 2). Targeting high sensitivity values of 90 and 95% as a screening test, using the logistic regression model could double specificity (17% vs. 33% and 10% vs. 20%), respectively, resulting in negative predictive values of 78% vs. 88% and 81% vs. 89%, respectively (Table 4).

**TABLE 4:** Specificity, PPV, NPV of current Rheport algorithm and best performing machine learning model according to targeted sensitivity.

	Current Rheport algorithm			Logistic regression		
		
Targeted sensitivity (%)	Specificity (%)	PPV (%)	NPV (%)	Specificity (%)	PPV (%)	NPV (%)
95	10	33	81	20	34	89
90	17	34	78	33	37	88
70	36	34	72	67	48	84

PPV, positive predictive value; NPV, negative predictive value.

### Feature importance

**FIGURE 3:**
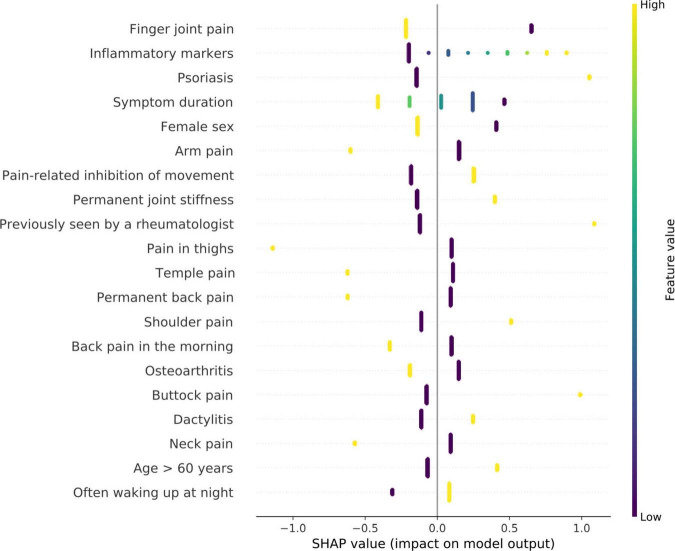
Feature importance of logistic regression model using SHAP values. High SHAP values (x-axis) represent a high impact on model output. negative values imply impact toward non-IRD classification and positive values direct toward classification as IRD. The y-axis describes the value levels; low representing 0 and negative answers.

The feature analysis included a total of 111 Rheport questionnaire features. Figure 3 lists the 20 most important features for the best performing model (logistic regression). The SHAP analysis reveiled finger joint pain, followed by inflammatory markers, psoriasis (as an underlying comorbidity), symptom duration and female sex as the five most important features. Reported finger joint pain directed the model toward Non-IRD classification, whereas underlying psoriasis and elevated inflammatory markers were top features directing toward IRD.

## Discussion

To our knowledge, we could show for the first time, that machine learning can improve the diagnostic accuracy of an online rheumatology referral system. The best ML model, namely logistic regression, promisingly improved the current Rheport algorithm (AUC of 0.737 vs. 0.543).

The subjectivity of current rheumatology triage decisions was recently highlighted by a study reporting an increase of inappropriate rheumatology referrals of 14.3% when a new rheumatologist was hired (20). The importance of pre-appointment management to improve efficacy of rheumatology consultations has been reported already by Harrington and Walsh (21). Having to make all prior patient records available for granting an appointment was key for a successful implementation. However, currently used hand written referral letters often lack this crucial information. Wong et al. recently reported that only 55% of referral letters included medical history, 51% laboratory parameters, and 34% imaging reports (22), depriving rheumatologists of crucial information. Deprived of information on prior external imaging and laboratory workup, even experienced rheumatologists are not able to correctly classify patients as IRD or non-IRD in the majority of cases (23). The most impactful features of the logistic regression model also highlight the need for having access to prior medical records and complete patient information, including current symptoms and basic laboratory parameters. Digitalization can significantly reduce the burden of making this information available prior to an appointment. In our opinion, digitally supported online rheumatology referrals could standardize and significantly improve triage decisions. The ability to communicate with referring physicians and to give them feedback could further improve referrals (21) and allow case resolutions after e-consultations, saving face-to-face visits of up to 20% (24). Displaying top feature importance using heat maps additional to the final Rheport score could effectively support rheumatologists in making an informed triage decision. Similarly, rheumatologists might be reluctant to implement a triage software, if feature importance cannot be adapted according the specific center.

Importantly the feature analysis reveiled some counterintuitive differences. Whereas increased inflammatory markers lead to an IRD classification, presence of finger joint pain lead to a non-IRD classification. One reason for this could be high proportion of patients with osteoarthritis (37.2%).

To improve the performance of Rheport, we believe that adding mandatory laboratory parameters and imaging results, similar to what rheumatologists need to make a correct decision (23), is vital. Solely relying on patient-reported symptoms, the accuracy of symptom checkers is relatively poor regarding correct IRD detection, with a sensitivity ranging between 14% (25), 19% (26, 27), and 54% (4). In a first analysis, comparing different symptom checker in rheumatology, including 34 patients, Powley et al. (27) showed only 19% patients with “inflammatory arthritis” were given the diagnosis of RA or PsA. Similarly, Proft et al. demonstrated, that using an online symptom checker- based self-referral tool for axial spondyloarthritis patients resulted in a correctly identified proportion of 19.4% (26). In a previous single-center study (4), we compared Rheport to an artificial intelligence-based symptom checker Ada, resulting in a similarly limited sensitivity and specificity of 53.7, 51.8, and 53.7 and 63.6%, respectively.

A strength of the study is the real-world and multicenter nature and size of the dataset used. A strength of Rheport is that only rheumatologists can add their final diagnosis. This represents extra work for the participating rheumatologists but adds high quality data, enabling a continuous improvement of the system (7). This study has some limitations. The majority of patients did not report any laboratory results. This might have influenced the results, especially regarding the high feature importance. Schneider et al. recently reported the added value of including laboratory parameters for machine learning based classification of inflammatory bowel disease in children (9). The high feature importance of inflammatory markers suggests that adding additional laboratory data such as antibody status could improve the accuracy of Rheport. As patients seem eager to use self-sampling (28, 29), future research should investigate the integration of self-sampling into online symptom checkers/self-referral tools. In a next study we will analyze whether it makes a difference if the information is entered by patients themselves or referring physicians. Despite the multicenter nature, the results are limited to one country and further validation studies are needed. Especially for more rare IRDs, also only scarcely included in the dataset, the accuracy is likely to be worse. New patient data and matching final diagnoses enable perpetual improvement of the algorithm. Implementing machine learning predictive models into clinical routine raises additional questions such as trust and liability.

## Conclusion

This study suggests that machine learning models can improve the diagnostic accuracy of online self-referral systems, based on patient reported data. Current accuracy is limited by the predominantly patient-reported subjective data. Routine collection of electronic patient-reported data of newly referred patients could enable an improved and standardized rheumatology triage strategy.

## Data availability statement

The raw data supporting the conclusions of this article will be made available by the authors, without undue reservation.

## Ethics statement

Ethical review and approval was not required for the study on human participants in accordance with the local legislation and institutional requirements. The patients/participants provided their written informed consent to participate in this study.

## Author contributions

JK, FK, PB-B, and MW: conceptualization. JK, FK, and LJ: methodology and formal analysis. JK, LJ, and CD: data curation. LJ and JK: writing—original draft preparation. JK, FK, CD, SK, WV, AK, DS, AH, FM, NV, GS, BE, MW, and PB-B: writing—review and editing. LJ: visualization. GS, NV, BE, PB-B, and MW: supervision. MW: funding acquisition. All authors have read and agreed to the published version of the manuscript.

## Conflict of interest

Qinum and RheumaDatenRhePort developed and hold rights for Rheport. WV, CD, and PB-B were involved in the development of Rheport. JK was a member of the scientific board of RheumaDatenRhePort. WV, CD, SK, PB-B, and MW were members of RheumaDatenRhePort GbR. RHADAR GbR received honoraria from UCB Pharma GmbH, Sandoz Deutschland/Hexal AG, Lilly GmbH, and Galapagos Biopharma Germany GmbH, and research support from Novartis Pharma GmbH. The remaining authors declare that the research was conducted in the absence of any commercial or financial relationships that could be construed as a potential conflict of interest.

## Publisher’s note

All claims expressed in this article are solely those of the authors and do not necessarily represent those of their affiliated organizations, or those of the publisher, the editors and the reviewers. Any product that may be evaluated in this article, or claim that may be made by its manufacturer, is not guaranteed or endorsed by the publisher.

## Footnotes

1
www.rheport.de
