# Supplementary material for: Machine learning-based improvement of an online rheumatology referral and triage system
Source: Front Med (Lausanne). 2022 Jul 22;9:954056. doi: 10.3389/fmed.2022.954056 (PMC9354580; doi:10.3389/fmed.2022.954056)
Supplement: Supplementary file 1 [file Table_1.DOCX]

**Table S2**. Rheport questionnaire responses.

| **Question** | **Response** |
| --- | --- |
| **Gender, n (%)** | |
| Female | 1570 (69.3) |
| Male | 695 (30.7) |
| **Age, n (%)** |  |
| <60 | 1741 (76.9) |
| >60 | 524 (23.1) |
| **Weight loss, n (%)** | |
| Not present | 1788 (78.9) |
| 2kg in 3 months | 140 (6.2) |
| 2-5kg in 3 months | 198 (8.7) |
| >5kg in 3 months | 139 (6.1) |
| **Duration of complaints, n (%)** | |
| < 6 weeks | 204 (9.0) |
| > 6 weeks, < 6 months | 723 (31.9) |
| > 6 months, < 12 months | 509 (22.5) |
| > 12 months | 359 (15.8) |
| couple of years | 470 (20.8) |
| **Preceding injury, n (%)** | |
| Yes | 139 (6.1) |
| No | 2126 (93.9) |
| **Preceeding infection*, n (%)** | |
| Soar throat or cold | 168 (7.4) |
| Pneumonia | 202 (8.9) |
| Diarrhea | 135 (6.0) |
| Vomitting | 37 (1.6) |
| Cystitis | 97 (4.3) |
| Urethritis | 32 (1.4) |
| **Preceeding tick sting, n (%)** | |
| Not present | 1997 (88.2) |
| During the last 6 months | 30 (1.3) |
| > 6 months, < 12 months | 47 (2.1) |
| > 12 months < 5 years | 191 (8.4) |
| **Previously seen physician(s)*, n (%)** | |
| General physician | 1928 (85.1) |
| Internal medicine | 168 (7.4) |
| othopedics | 1145 (50.6) |
| rheumatology | 192 (8.5) |
| other | 37 (1.6) |
| **Lab results, n (%)** | |
| Not available | 1426 (63.0) |
| CRP < 5 mg/l | 341 (15.0) |
| CRP > 5 mg/l < 10mgl | 170 (7.5) |
| CRP > 10 mg/l < 30mgl | 180 (7.9) |
| CRP > 30mgl | 90 (4.0) |
| ESR < 20 mm/h | 25 (1.1) |
| ESR > 20 mm/h < 35 mm/h | 15 (0.6) |
| ESR > 35 mm/h < 50 mm/h | 8 (0.4) |
| ESR > 50 mm/h | 10 (0.4) |
| **Family history, n (%)** | |
| Positive | 949 (41.9) |
| Negative | 1316 (58.1) |
| **Joint pain*, n (%)** | |
| In the morning | 1545 (68.2) |
| In the evening | 0 |
| At night | 0 |
| Whole day | 1545 (68.2) |
| At rest | 1739 (76.8) |
| During movement | 1781 (78.6) |
| In fingers | 1621 (71.6) |
| When shaking hands | 130 (5.7) |
| **Joint swelling, n (%)** | |
| Not present | 846 (37.4) |
| Hands (Picture RA) | 271 (12.0) |
| Big toe | 218 (9.6) |
| Hands (Picture Puffy) | 208 (9.2) |
| Big toe (Picture Osteoarthritis) | 185 (8.2) |
| Knee (Picture Osteoarthritis A) | 160 (7.1) |
| Knee (Picture Osteoarthritis B) | 156 (6.9) |
| Hands (Picture PsA) | 144 (6.4) |
| Hands (Picture Osteoarthritis) | 77 (3.4) |
| **Duration of joint swelling, n (%)** | |
| Not present | 731 (32.3) |
| sudden onset, duration of only 1-2 days | 498 (22.0) |
| > 6 weeks, < 6 months | 562 (24.8) |
| > 6 months, < 12 months | 194 (8.6) |
| > 12 months | 280 (12.4) |
| **Joint stiffness, n (%)** | |
| Not present | 484 (21.4) |
| Whole day | 583 (25.7) |
| Hands/Feet <30 min | 260 (11.5) |
| Hands/Feet >30 min <60 min | 290 (12.8) |
| Hands/Feet >60 min | 248 (10.9) |
| Shoulders/Hips <60min | 161 (7.1) |
| Shoulders/Hips >60min | 239 (10.6) |
| **Headache*, n (%)** | |
| At night | 109 (4.8) |
| At the temples | 334 (14.7) |
| Neck | 261 (11.5) |
| when chewing | 13 (0.6) |
| **Back pain*, n (%)** | |
| In the morning | 707 (31.2) |
| In the evening | 80 (3.5) |
| At night | 110 (4.9) |
| Whole day | 367 (16.2) |
| At rest | 66 (2.9) |
| Worsening with movement | 141 (6.2) |
| Improvement with movement | 61 (2.7) |
| **Other pain*, n (%)** | |
| Often everywhere | 36 (1.6) |
| Most of the time | 51 (2.3) |
| Worsening with cold temperature | 41 (4.8) |
| Sudden onset | 138 (6.1) |
| Slow onset | 33 (1.5) |
| Neck | 245 (10.8) |
| Shoulders | 362 (16.0) |
| Arms | 339 (15.0) |
| Back | 300 (13.2) |
| Buttocks | 185 (8.2) |
| Thighs | 189 (8.3) |
| Stiffness | 635 (28.0) |
| Morning stiffness | 82 (3.6) |
| **Pain related inhibition of movement, n (%)** | |
| Yes | 994 (43.9) |
| No | 1271 (56.1) |
| **Muscle weakness*, n (%)** | |
| Lifting arms | 997 (44.0) |
| Rising from chair | 752 (33.2) |
| Climbing stairs | 782 (34.5) |
| **General symptoms*, n (%)** | |
| Feeling sick | 179 (7.9) |
| Often wake up at night | 1784 (78.8) |
| Sleepdeprived | 1623 (71.1) |
| Tired | 1812 (80.0) |
| No appetite | 188 (8.3) |
| **Other symptoms*, n (%)** | |
| Fever without specific reason | 119 (5.3) |
| Burning dry eyes | 791 (34.9) |
| Dry mouth | 816 (36.0) |
| Swallowing problems | 377 (16.6) |
| Change of colour of fingers or toes | 377 (16.6) |
| Sun allergy | 212 (9.4) |
| Skin problems | 278 (12.3) |
| Cold body | 112 (4.9) |
| Often diarrhea | 470 (20.8) |
| Trouble with breathing | 850 (37.5) |
| **Comorbidities*, n (%)** | |
| Psoriasis | 287 (12.7) |
| Hyperuricemia | 208 (9.2) |
| Obesity | 714 (31.5) |
| Osteoarthritis | 843 (37.2) |
| Liver disease | 49 (2.2) |
| Pulmonary disease | 251 (11.1) |
| Diabetes | 151 (6.7) |
| Thrombosis | 102 (4.5) |
| Previous misscariage | 171 (7.5) |
| Inflammatory bowel disease | 116 (5.1) |
| Renal disease | 99 (4.4) |
| Thyroid disease | 489 (21.6) |
| Depression | 425 (18.8) |

*multiple answers could be given.
